# Pilot study: feasibility of using the Suicidal Ideation Questionnaire (SIQ) during acute suicidal crisis

**DOI:** 10.1186/1753-2000-8-28

**Published:** 2014-11-04

**Authors:** Isabel Boege, Nicole Corpus, Renate Schepker, Joerg M Fegert

**Affiliations:** ZfP Südwürttemberg, Abteilung für Psychiatrie und Psychotherapie des Kindes- und Jugendalters, Weingartshoferstrasse 2, 88214 Ravensburg, Germany; Universität Ulm, Klinik für Kinder und Jugendpsychiatrie und Psychotherapie, Ulm, Germany

**Keywords:** Suicidal ideation, Suicidal ideation questionnaire, Suicide risk assessment, Suicidality, Youth-life status questionnaire

## Abstract

**Background:**

Assessing youths in acute suicidal crisis is a common jet pivotal task in child and adolescent psychiatry, usually relying primarily on the clinicians skills of assessment. The objective of this pilot-study was to evaluate feasibility and usefulness of questionnaires during assessment of youths in acute suicidal crisis.

**Method:**

31 adolescents, presenting for suicide assessment, and their caregivers, were asked upon emergency presentation to fill in the Suicidal-Ideation-Questionnaire (SIQ) and the Youth Life Status Questionnaire (Y-LSQ) before receiving an assessment by a clinician. The SIQ has 30 items, 8 of which are defined as critical items able to predict suicidality with the highest probability. The Y-LSQ (30 items) measures the overall level of psychological distress. It has one suicidal item, which was used in this study for validation of the SIQ result. Clinical judgment and test results were collected and analyzed by an independent researcher.

**Results:**

It was feasible to ask adolescents in acute suicidal crisis to fill in a questionnaire. Clinical assessment of suicidality did not correlate significantly with the overall SIQ-score (p = 0.089), however there was a significant correlation between the SIQ 8 critical item result and clinical judgement of suicidality (p = 0.050).

**Conclusion:**

The 8 critical SIQ items can be used to support clinical judgment of suicidality in acute crisis.

## Background

Suicide is the second to third leading cause of death in adolescents aged 15 to 24 years [1, 2]. Risk-assessment in youths presenting with suicidal ideation is therefore a frequent and pivotal task in child and adolescent psychiatry. Reported self-harm, suicidal ideation, and previous suicide attempts have to be taken seriously as they are most highly associated with later suicide, prior attempts being the most predictive stable factor; plans and preparations the most predictive accurate factor [3]. Suicidal ideation therefore has to be always assessed thoroughly [4–6]. Females are more likely to report suicidal ideation and behaviour [7], while males are more likely to complete suicide [8].

Many risk factors have been identified in the past: Personal factors [9], family factors [9, 10], presence of mental illness [11]. However a specific set of symptoms that most accurately predicts suicidal behaviour has not yet been found [12].

The use of standardized methods during assessment to classify the spectrum of suicidal ideation and behaviour as well as risk factors present is recommended [13] but not always routinely done.

A vast array of instruments have been designed to measure various aspects of suicidal ideation, acute risk of suicide and differentiate non suicidal selfharm from selfharm with suicidal intent. Well assessed screening instruments are available (e.g. Selfharm Behaviour Questionnaire [14], Columbia Suicide Screen [15], The Risk of Suicide Questionnaire, Suicide Risk Screen [16], Suicide Probability Scale [17, 18]). However these instruments bear several limitations which have been discussed in the literature: Instruments measuring aspects of suicidality are known for their high false positive rate. Some use static variables (eg family history) that do not change over time, possibly underestimating the acute level of exacerbation. Predictive validity for most suicide measures has not been established. Brief screening instruments have been mainly developed for and assessed in research populations, making their generalizability for the regular primary care setting questionable [19].

Risk-assessment for suicidal youths therefore remains to be a difficult clinical task [20].

The objective of this pilot study was to investigate the feasibility, significance and implication of routinely using suicidal-ideation-questionnaires during assessment of the suicidal youth. The underlying hypotheses to be tested were: (1) Youths will fill in the questionnaires; (2) clinical assessment will correlate with the SIQ score (3); the suicide item on the Y-LSQ will correlate with the SIQ score.

## Methods

### Sample

Participants were 35 adolescents presenting consecutively for emergency assessment of suicidality between May 2010 and January 2011 to the department of child and adolescent psychiatry, ZfP Suedwuerttemberg, Germany. The department of Child and Adolescent Psychiatry serves a catchment area of 600.000 inhabitants. A 24 h/7d child and adolescent psychiatric service for the whole range of psychiatric crises is available. The mean age in the sample was 15.7 years (SD =1.09). Of the included adolescents 13 were male (41,9%) and 18 female (58.1%). Main reason for referral was suicidal ideation, attempted suicide and selfharm.

### Instruments

#### Suicidal Ideation Questionnaire (SIQ)

The SIQ [21] is a self-report instrument for suicidal ideation, appropriate for ages 14.0 to 17.11. As one component in a comprehensive assessment of adolescent mental health it can serve the professional as an initial source of information. It does not predict suicide in itself [18], however it has been shown to be a moderately to highly sensitive marker of possible subsequent suicide attempts and broad suicidality [22]. It has a 98% sensitivity, 37% specificity, and a 55% positive predictive value [23]. For the total SIQ standardization sample (n = 890) internal consistency reliability estimates rank uniformly high from .969 to .974, with a total sample reliability coefficient of .971. [21, 24, 25]. Content validity for the SIQ items ranges from .70 to .90, with a median correlation of .78 for the total sample.

The SIQ has 30 items, ranging from very minor/nonspecific thoughts (e.g. I wish I was never born) to major/specific thoughts (e.g. I thought of when I would kill myself). Each item on the SIQ begins with “I thought…”, “I wondered…”, “I wished…”. The respondent is asked to choose from a 7 point continuum (between “Almost every day” to “I never had this thought”) to assess the frequency of that particular thought within the last month. A high score on the SIQ is indicative of frequent and pervasive suicidal ideation. Scores and items can be used in four basic ways: total score, cut-off scores, critical item review, or clinical perusal of individual items. Cut-off score for the SIQ is a sum of 41 and higher, indicating the need of further evaluation of psychopathology. 8 “critical items” are defined, which predict self-destructive behaviour best. If an adolescent scores a 5 or 6 on more than three of these items he/she is considered to be at higher risk for suicide irrespective of the total SIQ-score [21]. The 8 items are presented in Table 1.

**Table 1:** **Core items of the SIQ, that form the “scale of the 8 critical items”**

No	Item	6	5	4	3	2	1	0
3	I thought about how I would kill myself	○	○	○	○	○	○	○
4	I thought about when I would kill myself	○	○	○	○	○	○	○
5	I thought about people dying	○	○	○	○	○	○	○
7	I thought about what to write in a suicide note	○	○	○	○	○	○	○
8	I thought about writing a will	○	○	○	○	○	○	○
9	I thought about telling people I plan to kill myself	○	○	○	○	○	○	○
13	I thought about how easy it would be to end it all	○	○	○	○	○	○	○
18	I thought if I had the chance I would kill myself	○	○	○	○	○	○	○

0 = I never had this thought, 1 = I had this thought before, but not in the past month, 2 = About once a month, 3 = Couple of times a month, 4 = About once a week, 5 = Couple of times a week, 6 = Almost every day.

For the pilot study the SIQ was translated into German, a retranslation was preformed to ensure correctness of translation. To assure understandability further two questions querying understanding and straightforwardness of answers were added to the SIQ.

### Youth-Life Status Questionnaire (Y-SLQ)

The Y-LSQ [26] is designed to describe a wide range of situations, behaviors, and moods that are common to adolescents.

It is a 30-item tool assessing clinical risk and the patient’s overall level of psychological distress. Each item scores on a 5 point continuum between 0 (“never or almost never”) and 4 (“almost always or always”), giving a total score range between 0 and 120. The score is categorized into normal (0–38), mild (52–64), moderate (52–64) and severe psychological distress (65–120). Six subscales can be evaluated: somatic problems, social isolation, behavioral problems, aggression, hyperactivity and depression/anxiety. The Y-LSQ encompasses one suicide item, which was of interest for this study [27, 28]. It has a high reliability of 0.77-0.96 (Youth report) respective 0.92 (parent/carer report) and discriminates in its validity between clinical and community samples [29].

### Suicide Risk Checklist

The suicide-risk-checklist, resembles an adaptation of the semi-structured instrument “Tool for Assessment of Suicide Risk” (TASR). TASR is neither a diagnostic tool since suicide is a behaviour rather than a medical diagnosis nor a predictive tool as there exists no tool that has been shown to predict reliably suicide [12]. It is a standardized checklist which, embedded in a broader framework of assessment (e.g. mental status exam), allows professionals to assess the risk for youth suicide by following a standardized evaluation of the most common risk factors known to be associated with suicide in young people. Risk factors are grouped in (1) individual risk profile (e.g. male, age, family history…), (2) symptom risk profile (e.g. hopelessness, worthlessness, anger, impulsivity…) and (3) interview risk profile (e.g. suicidal ideation, attempted or planned suicide, recent alcohol/drug abuse, access to lethal means, unsolvable problems). Individual-risk-profile items weigh 1 point, symptom-risk-profile items 2 points and interview-risk-profile items 3 points. The score indicates high, medium or low suicide risk.

The questionnaires were translated from English into German, re-translation by a native speaker proved to be reliable for each item.

### Procedure

The study was conducted in compliance with the Helsinki Declaration and approved by the Ethical Committee of the University of Ulm (145/19) in August 2010. All clinicians working on-call were trained in administering the suicide-risk-checklist.

Inclusion criteria for participants were: (1) age ≥14; (2) primary reason for referral: assessment of suicidality or self-harm; (3) parent/carer present on site; (4) written informed consent of adolescents and their caregivers. Once enrolled participants and their carers completed - before they saw the clinician - the SIQ and Y-LSQ. After handing in the questionnaires to a nurse, standard psychiatric assessment for suicidality was performed by the clinician within 15–30 min (on average). Assessment lasted Ø 60 min, at the end of which the clinician filled in the suicide risk checklist. The classification of low, medium and high suicide risk was done by clinical judgement, supported by the suicide-risk-checklist. Clinicians were not aware of the SIQ or the Y-LSQ result during their assessment. The questionnaires and the standardized suicide-risk-checklist were analysed afterwards by an independent researcher.

### Statistical analysis

Data was analysed using SPSS version 21.0. Cronbach’s alpha was calculated to give an orientation for the internal consistency of items in der German SIQ version. Descriptive statistics were used for demographic data. Two logistic regression models were built to explore the predictive power for low or high/medium risk assessment of the SIQ score, Y-LSQ item and indication for admission as inpatient (model 1) or for the SIQ, 8 critical items score, Y-LSQ item and indication for admission as inpatient (model 2). In a third model, the predictive power of age, gender and Y-LSQ score for the SIQ score was tested.

## Results

Main findings of this study are presented in Table 2 (demographic data) and Table 3 (SIQ results).

**Table 2:** **Demographic data, clinical risk assessment and total SIQ score**

Suicide risk assessment (risk checklist & clinical judgement)		Low	Medium	High	Total
**n**		10	18	3	31
**Age (mean)**		15,7 (SD 1.6)	15,7 (SD 1.1)	16,3 (SD .6)	15,6 (SD 1.2)
**Female/male (n)**		4 / 6	12 / 6	1 / 2	54,8% / 45,2%
**Diagnoses (n)**	**F32**	4	5	0	26,5%
	**F41**	1	0	0	2,9%
	**F43**	1	6	0	23,5%
	**F60**	0	2	1	8,8%
	**F90**	0	0	1	2,9%
	**F91**	1	0	0	2,9%
	**F92**	4	3	1	26,5%
	**F94**	0	1	0	2,9%
**SIQ total (mean)**		53,54 (SD 39,7)	85,18 (SD: 46,1)	88,0 (SD: 36,5)	--
**Selfharm prior to assessment**		3/10 (30%)	11/18 (61%)	1/3 (33%)	15/31 (48,4%)

**Table 3:** **Mean and multivariable analysis for SIQ total and SIQ 8 item**

	Low risk (n = 10)		High risk (n = 21)	
**SIQ-range (max 180)**	0-119		0-151	
**SIQ 8 item range (max 42)**	0-23		0-47	
	**mean**	**SD**	**mean**	**SD**
**SIQ total**	48.34	38.271	89.61	41.041
**SIQ 8 item**	7.78	6.667	22.15	12.918
**Y-LSQ**	1.56	1.424	2.50	1.192
**model 1: dependent variable: high/medium versus low risk group ( adjusted R** ^**2**^ **= .382)**				
	**coefficient (95% CI)**		**p**	
**SIQ total**	.005(−.001 to .010)		.093	
**Y-LSQ Item**	-.048 (−.236 to .140)		.603	
**Admission as inpatient**	.588(.230 to .946)		.002	
**model 2: dependent variable: high/medium versus low risk group ( adjusted R** ^**2**^ **= .429)**				
	**coefficient (95% CI)**		**p**	
**SIQ 8 item**	.016(.002 to .031)		.029	
**Y-LSQ Item**	-.026(−.168 to .117)		.716	
**Admission as inpatient**	.557(.211 to .903)		.003	
**model 3: dependent variable: SIQ total ( adjusted R** ^**2**^ **= .646)**				
	**coefficient (95% CI)**		**p**	
**Y-LSQ Item**	24,958(16,529 to 33,386)		.000	
**Age**	1,497(−8,963 to 11,956)		.771	
**Gender**	15,797(−8,282 to 39,876)		.189	

### Demographic data

31 of 35 adolescents eligible participated. One adolescent did not meet the age criteria. Three youths refused (8.6%) to fill in the questionnaires, two of them, oppositional throughout, returned the questionnaires blank; one appeared to be too distressed to fill in a questionnaire. Assessment on the suicide-risk-checklist classified participants as *low risk* in n = 10, *medium risk* in n = 18 and *high risk* in n = 3 cases. Reasons for referral were: attempted suicide (12,9%), suicidal ideation (29%), threat of suicide (16,1%), non suicidal self injury (9,7%), self harm with suicidal intent (12,9%), other (eg alcohol intoxication) (19,4%). 48,4% presented with self harm in their history.

23 youths were admitted after assessment for crisis intervention (79,3%). Of those patients categorized as low risk group 40% (n = 4) were admitted while 90,5% (n = 19) of patients in the medium/high risk group were admitted. Discharge took place on average after 4.57 days (*SD* =3.59). 8 youths were discharged right after outpatient emergency assessment, receiving a follow-up appointment within one week after assessment.

Main ICD10 diagnoses given were: affective disorder (F32.1/F34.1) (29,4%), conduct disorder, mixed (F92.0) (26,5%) and adjustment disorder (F43.2) (23.5%) (Table 1). There was no correlation between high suicidality and a specific diagnosis.

### Questionnaires

The German SIQ had a Cronbach’s alpha of .97, the average item-intercorrelation was .53. All participants filled in the SIQ (Mdn = 76.80, SD =44.033, range: 0–151), while only 26 participants filled in the Y-LSQ (Mdn = 58.55, SD = 17.872, range 7–101). Most of the youths found all questions understandable (71%) and asserted that they answered straightforward (90,3%). Reasons for not understanding questions were not given.

### Main results

Due to small numbers the participants classified during assessment as medium and high risk were summarized for further calculations as one group. Low risk participants reported lower scores on the SIQ (Mdn = 48.34), while medium/high risk participants reported higher scores (Mdn = 89.61). The total SIQ score did not correlate significantly with clinical risk assessment (p = .093). But the score on the “8 critical SIQ-items” correlated significantly with the clinical-risk-assessment (p = .029): Youths with a low risk-assessment (Mdn =7.78) scored significantly lower on the 8 critical items than youths with a medium/high risk-assessment (Mdn =22.15).

The Y-LSQ suicide item (“I think about suicide or feel I would be better off dead”) and the SIQ result correlated significantly both on the SIQ total score as well as on the SIQ 8 critical item score (p = .000). (Table 3)

## Discussion

This study indicates that the SIQ can be used during clinical assessment of adolescents in crisis. Youths will fill in questionnaires before meeting the clinician. Only 3 youths refused to participate, due to underlying symptomatic (distress, overall oppositional behaviour). That is far below the quota of 20%, which still would be acceptable.

With an internal consistency of .97 and an average inter-item correlation coefficient of .53 the German SIQ version seems to reproduce the internal consistency and inter-item correlation coefficient of the English instrument [30]. The significant correlation of the Y-SLQ suicide-item with the SIQ-score underlines the construct validity of the SIQ and enhances the findings of the original research study [21, 24]. However due to the small sample size this may only be a figure for orientation. A reliable estimation needs a larger sample. In a larger study, it also should be of interest, which questions youths find difficult to understand. The majority of adolescents stated that they answered truthfully, but only 71% asserted that they found all questions understandable. Amount of distress, symptoms associated with certain diagnoses (eg schizophrenia, autism) or wording of the items are possible explanations and should be differentiated in further studies.

The reason for referral in this sample was suicidality or self-harm, therefore the intention, when handing out the SIQ, was not to screen for suicidal ideation. However, risk assessment in suicidal youths is complex. A thorough risk assessment should therefore include several sources of information. Youths may not disclose all relevant information in an interview. We queried if a) youths are generally able to fill in a questionnaire in acute crisis and b) if information given on the SIQ questionnaire reliably affirms clinical judgement. The lack of correlation between the total SIQ score and the clinical risk assessment was surprising, but may be explainable by several facts: (a) the small sample size; (b) different points in time of reference: the SIQ covers suicidal ideation within the past month, whilst the suicide-risk-checklist assesses suicidality at emergency presentation; (c) time at which information is given: the SIQ is filled in *before* assessment, the suicide-risk-checklist *after* assessment, about 1.5 hours later, when the actual risk of suicidality may have already decreased; (d) self-report data may differ from information gained in an interview: Some studies report generally high correspondence of these two sources of information [31] others depict low concordance [5, 32]. Safer et al. point out that reports of suicidal ideation are up to 2–3 times more likely, when retrieved via questionnaires than via interview [33]. Other studies report that suicidal ideation fluctuates within short periods of time [34]. All of them stress the importance of using different approaches to measures the risk of suicidality accurately.

When analysing only the 8 critical items of the SIQ in correlation with clinical assessment the result changes: the score of the 8 critical items on the SIQ correlates significantly with clinical risk-assessment. This goes conform to a study from Gutierrez and Osman [35] who demonstrated in a large high-school as well as in a clinical sample that the 8 critical items perform well in differentiating suicide attempters from non suicidal high-school students.

### Limitations

Methodological limitations to be noted are: All youths, who were included, presented for assessment of suicidality, the absence of non-suicidal individuals filling in the questionnaire may have biased the results. Also, due to the small sample, the significance of the findings is limited. The findings should be reproduced in a larger mixed sample, comparing a school sample (in which a lower rate of suicidal ideation is to be expected) with a clinical sample, including all reasons for referral (with probably a higher rate of suicidal ideation), to validate the results. Sensitivity and specificity should be also evaluated for subpopulations such as restrained youths.

In addition, it has to be taken into account that youths included were emergency presentations and evaluated by the clinician on call. The heterogeneity of experience and training of the clinicians may have caused a non-homogeneous risk assessment.

## Conclusions

Risk assessment in suicidal youths is complex. A thorough assessment should include several sources of information. The SIQ, especially the 8 critical items, which correlate well with clinical assessment, is a feasible instrument for youths in acute crisis. With caution it can be concluded that using the SIQ during assessment can complement but not replace clinical assessment. Larger samples are needed.
